# Bayesian learning from multi-way EEG feedback for robot navigation and target identification

**DOI:** 10.1038/s41598-023-44077-8

**Published:** 2023-10-07

**Authors:** Christopher Wirth, Jake Toth, Mahnaz Arvaneh

**Affiliations:** 1 Automatic Control and Systems Engineering Department, University of Sheffield, Sheffield, S1 4DT UK; 2 School of Medical Sciences, University of Manchester, Manchester, M13 9NT UK

**Keywords:** Learning algorithms, Neural decoding, Neurophysiology

## Abstract

Many brain-computer interfaces require a high mental workload. Recent research has shown that this could be greatly alleviated through machine learning, inferring user intentions via reactive brain responses. These signals are generated spontaneously while users merely observe assistive robots performing tasks. Using reactive brain signals, existing studies have addressed robot navigation tasks with a very limited number of potential target locations. Moreover, they use only binary, error-vs-correct classification of robot actions, leaving more detailed information unutilised. In this study a virtual robot had to navigate towards, and identify, target locations in both small and large grids, wherein any location could be the target. For the first time, we apply a system utilising detailed EEG information: 4-way classification of movements is performed, including specific information regarding when the target is reached. Additionally, we classify whether targets are correctly identified. Our proposed Bayesian strategy infers the most likely target location from the brain’s responses. The experimental results show that our novel use of detailed information facilitates a more efficient and robust system than the state-of-the-art. Furthermore, unlike state-of-the-art approaches, we show scalability of our proposed approach: By tuning parameters appropriately, our strategy correctly identifies 98% of targets, even in large search spaces.

## Introduction

Brain-computer interfaces (BCIs) read and interpret signals directly from the brain, allowing severely disabled people the possibility of controlling assistive robots^1–4^. There is a performance bottleneck in many BCIs, as users are required to control each low-level action in order to achieve a high-level goal. For example, users may need to consciously generate brain signals to move a cursor, prosthesis, or assistive robot, step-by-step to a desired location. Indeed, BCI navigation systems have existed for wheelchairs and robots for well over a decade^5–7^. However, these systems generally require the user to take active control of individual actions such as each change in direction. This places a high mental workload on the user.

Machine learning provides the potential to alleviate this mental burden. Recent studies have shown the possibility of using “cognitive probing” - monitoring reactive brain signals in response to certain machine actions^8^, and using these signals as feedback for reinforcement learning (RL), thus allowing robots to learn to perform tasks^9,10^. These studies have mostly been based on distinguishing correct actions from erroneous ones, by detecting error-related potentials (ErrP) - characteristic signals that are spontaneously produced in the brain in response to an error recognised by the human^11,12^. Indeed, previous studies achieved encouraging results for robot path planning using ErrP detection combined with RL^13–17^. However, there are other options for route planning without the need for human intervention. In outdoor scenarios, satellite navigation is available. For smaller scale or indoor scenarios, there are myriad methods for robot path planning without the need for information from EEG^18–23^. One thing that cannot be inferred without input from the human, is which target they wish the robot to travel towards. Arguably, therefore, “target identification” is the more important piece of information to learn from the EEG.

A few studies have begun to tackle this problem. Chavarriaga and Millán used EEG-based RL to choose between whether a target was on the left or the right of the machine’s current location^24^. Some have indicated the possibility of using EEG rewards to infer which specific location, from a subset on a grid, was the target. In a recent study, Schiatti et al. had the machine converge upon optimal routes to each potential target via Q-learning, and then implemented a second layer of Q-learning to choose between possible targets^16^. However, in this paradigm, the robot received an environmental reward and stopped automatically when the target was reached, assuming that there was an external way to know the target was correct, rather than inferring this information purely from EEG responses to the observed actions. Iturrate et al. showed that it is possible to converge on targets without the need for such external validation - their approach similarly used Q-learning to find optimal routes, and then compared feedback rewards with expected Q-values^13^. However, it has been stated that Q-learning suffers from poor scalability^14^ and, indeed, these studies each had only a small subset of potential target loci in relatively small areas. Iturrate et al. recently stated, “It is then an open question how the proposed [brain-machine interface] paradigm may generalize across tasks or scale to more complex scenarios”^14^. Furthermore, all of these studies used binary classification of robotic actions, classifying each movement simply as correct or erroneous.

A small number of recent studies have shown that it is feasible to obtain more detailed information from reactive brain signals than simply whether an action was correct or erroneous^9,25^. Building on this, our recent work showed that it is possible to subclassify different types of navigational errors against each other^26^, and to subclassify different correct navigational actions against each other^27^. Most recently, we showed the possibility of performing a detailed 4-way single-trial EEG classification of all of these navigational actions^28^.

This paper, for the first time, proposes 4-way EEG-based learning for robot navigation and target identification, rather than binary EEG classification used in previous studies. The 4 classes of movements were: towards the target but not reaching it, reaching the target, moving from an off-target location to a location even further away from the target, and stepping directly off the target location. In this study, the user’s intended target will be inferred from reactive brain signals, generated spontaneously as the user merely observes the robot’s actions. Importantly, the target could be any location in the space.

In order to infer the target location, a machine learning system is implemented. In this case, we observe that the task can be framed as a probabilistic problem. We therefore propose a learning strategy of Bayesian inference, utilising prior knowledge of classification contingency tables to contextualise EEG responses, and build a probabilistic model in order to learn the most likely target location. This allows for a simple, scalable solution. Furthermore, as we are making the reasonable assumption that the path from one location to another does not need to be learned via EEG, this system is inherently faster than state-of-the-art methods requiring EEG-based route planning, in which a process (e.g. Q-learning) must be undertaken to learn the map, before target acquisition can begin.

In order to test both efficiency and scalability, we investigate our strategy’s effectiveness in both small ($9\times 1$, i.e. 9 spaces) and large ($20\times 20$, i.e. 400 spaces) grids using EEG data recorded from 10 participants. Furthermore, we investigate the trade-off between speed and accuracy, using an adjustable parameter to control the level of evidence that must be accumulated before converging on a particular target.

Our proposed methodology moves the research area forward in two key ways:

1. The use of more detailed EEG information allows the possibility of more efficient learning.
2. The Bayesian learning method, assuming a known map of possible locations, allows the system to be scalable.

## Experimental design

### Dataset

**Figure 1:**
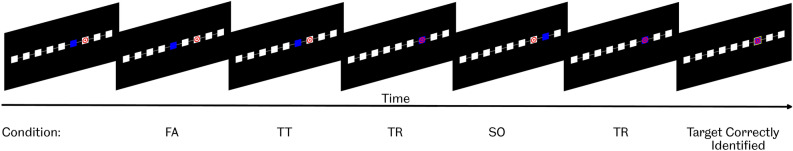
The experimental paradigm observed by participants. Stills are shown from a subsection of a single run. The 9 × 1 grid is shown. In the first (leftmost) still, the blue cursor is in position 6, and the target - denoted by a bullseye symbol - is one place to the right in position 7. In the second still, the robot moves one step to the left, to position 5. This is a move further away from the target (FA condition). Following this, in the third still, the robot moves back from position 5 to position 6 - this is a move towards the target (TT condition). In the fourth still, the robot moves right from position 6 to position 7. As the target is in position 7, this is a move in which the target is reached (TR condition). However, in the fifth still, the robot continues to move right, and steps off the target (SO condition) to position 8. In the sixth still, the robot moves left, back to position 7 - a move that once again reaches the target (TR condition). Finally, in the seventh still, the robot correctly identifies the target by drawing a yellow box around its current location, which is the target locus. It is also possible for incorrect loci to be erroneously identified as the target.

In the present study, we used real EEG data from our previous work^27,28^. Ten healthy adults (4 female, 6 male, mean age 27.30 ± 8.31) had merely observed a virtual robot navigation paradigm. Written informed consent was provided by all participants before testing began. All procedures were in accordance with the Declaration of Helsinki, and were approved by the University of Sheffield Ethics Committee in the Automatic Control and Systems Engineering Department. The brain signals were recorded at 500Hz, at electrode positions Fz, Cz, Oz, Pz, C3, C4, PO7, and PO8, using an Enobio 8 headset. The paradigm consisted of a cursor, representing the virtual robot, moving left and right in a 1-dimensional space on a screen, made up of 9 locations, as shown in Fig. 1. The robot’s stated goal was to reach, and correctly identify, a target location. However, erroneous movements (such as that illustrated in Fig. 1) and erroneous target identifications occurred with preset probabilities.

The target symbol was placed in a random location in the grid at the beginning of each run. The cursor, represented by a blue square, was placed 2 or 3 steps away, either on the left or right. Every 2 seconds, the robot performed one of the following actions: If the robot was not currently on the target, there was a 70% chance it would move towards the target (this would result in reaching the target if it started 1 step away), a 20% chance the robot would move further from the target, and a 10% chance the robot would erroneously identify its current location as the target, by drawing a yellow box around it. If the robot was on the target, there was a 67% chance it would correctly identify its location as the target by drawing the yellow box, but a 33% chance it would step off the target.

Each run ended as soon as the robot identified a location as the target, whether this was correct or erroneous. There was then a 5 second period in which the screen was blank before the next run began. Participants were told that they could move and blink freely in the interim periods, but asked to refrain from movement or blinking during runs. Runs continued in this manner for blocks of approximately 4 minutes. Participants were given inter-block breaks of as long as they chose, and observed blocks until they reported a reduction in concentration. Eight participants observed six blocks each, and a further two participants observed two blocks each.

### Workflow

As the classification and simulations in this study were subject-specific, we can consider the workflow for each participant to be independent of the other participants. With this in mind, the steps taken for a given participant were as follows:

- Observation: the participant observed the experimental paradigm. Their EEG responses to the four different movement action conditions, and two different target identification conditions (correct and false targets), were recorded.
- EEG data preprocessing: EEG trials of each different class were preprocessed.
- Training and test data separation: Within each class, 85% of trials were randomly selected as the training set. The remaining 15% of trials were designated as the test set.
- Training the classifiers: The participant’s training trials were used to build classification models for 4-way classification of movement actions, and binary classification of target identification actions.
- Simulations: Offline simulations were run. In these simulations, after each action was performed by the robot, a trial was retrieved from the participant’s appropriate test set (i.e. an EEG response to the same class of action, as previously observed by the participant). The classification of these trials provided information that the navigation strategies could use to guide them toward the correct target.

We expand upon each of these steps in the coming sections.

### Simulations using real EEG

The goal of this study was to simulate a real-time implicit human-machine interaction. In order to achieve this, while being able to explore a variety of scenarios, running through each a large number of times, we used previously-recorded real EEG data as feedback for the robot. This approach has recently proven useful in exploratory studies^29^.

Of the ten participants who observed the paradigm, two did not produce enough artefact-free trials in each of the four movement classes, and were excluded. Therefore, simulations were performed using EEG generated by the remaining eight participants. Subject-specific classifiers were trained. For this purpose, 85% of each participant’s EEG trials from each class were randomly selected as training samples, with the remaining 15% being reserved as test samples. The number of training and test trials in each class, for each participant’s model, are shown in Supplementary Tables 1–4.

In the simulation phase, two different grids were used. Firstly, the virtual robot navigated in a 1-dimensional space made up of 9 squares. Secondly, the virtual robot navigated in a 2-dimensional space made up of 400 squares, arranged in a 20$\times$20 square. The EEG data were originally recorded in an offline session, while participants observed the 1-dimensional version of this paradigm, as described in the above subsection, ‘Dataset’. The 2-dimensional version was designed such that the same movement classifications (as described in further detail in the Methods section, ‘Multi-way classification of robotic actions’) remained valid, i.e. it was not possible for the robot to perform a move that resulted in it remaining the same distance from the target. The target could be randomly positioned in any location (i.e. any square in the grid) at the start of each run. The robot began the run at a randomly selected location a minimum of 2 moves away from the target, with no maximum distance imposed other than the boundaries of the space. Each action consisted of either (a) a discrete movement from the robot’s current square to an adjacent square, or (b) identifying the robot’s current square as the target location.

Three navigation strategies were tested, namely our proposed “Bayesian Inference”, “Random”, and “React”. The precise details of these navigation strategies are described in the Methods section, ‘Navigation strategies’. For all strategies, at the start of each run, an initial action was selected at random. Then, for the “React” and “Bayesian Inference” strategies, after each action was performed by the robot, a test EEG trial from a participant who had previously observed the experiment was retrieved. The trial was selected randomly from the test trials of the appropriate class, e.g. if the robot had moved towards the target, the trial would be one which was recorded when the participant had, similarly, observed the robot moving towards the target. The trial was then classified as if it were being processed in real time (as described in the Methods section, ‘Multi-way classification of robotic actions’). The classification output provided by the trained model was used to inform future actions of the robot (as described in the Methods section, ‘Navigation strategies’). Note that, as in any EEG study, some trials were misclassified. Test data classification accuracy, for each class and each participant, is shown in Supplementary Tables 5, 6, and detailed contingency tables of these classifications are shown in Supplementary Tables 7–38. The process described here continued, using one randomly selected pre-observed EEG trial from the same participant after each robot action, until the navigation strategy determined that the target had been reached. At this stage, the run ended, and statistics were recorded denoting the number of steps the robot had taken during the course of the run, and whether the correct target location had been identified. Each navigation strategy was run 1000 times per participant on each grid size.

## Methods

### Multi-way classification of robotic actions

For each participant, a four-way classification model was trained to distinguish four classes of observed movement:

- “TT condition”: Towards target (but not reaching it),
- “TR condition”: Target reached,
- “FA condition”: Further away (when moving from an already off-target location, to a location further away from the target),
- “SO condition”: Stepped off target.

Distance to the target was calculated as the minimum possible number of steps from the robot’s current position to the target. The robot could not move diagonally. Therefore, for example, from position [2, 2] in a 2-dimensional grid, with a target at position [5, 5], the distance to the target would be 6 steps (3 horizontal + 3 vertical). From this position, there would be two possible “TT condition” movements (to [2, 3] or [3, 2], each reducing the distance to 5 steps), and two possible “FA condition” movements (to [2, 1] or [1, 2], each increasing the distance to 7 steps).

**Figure 2:**
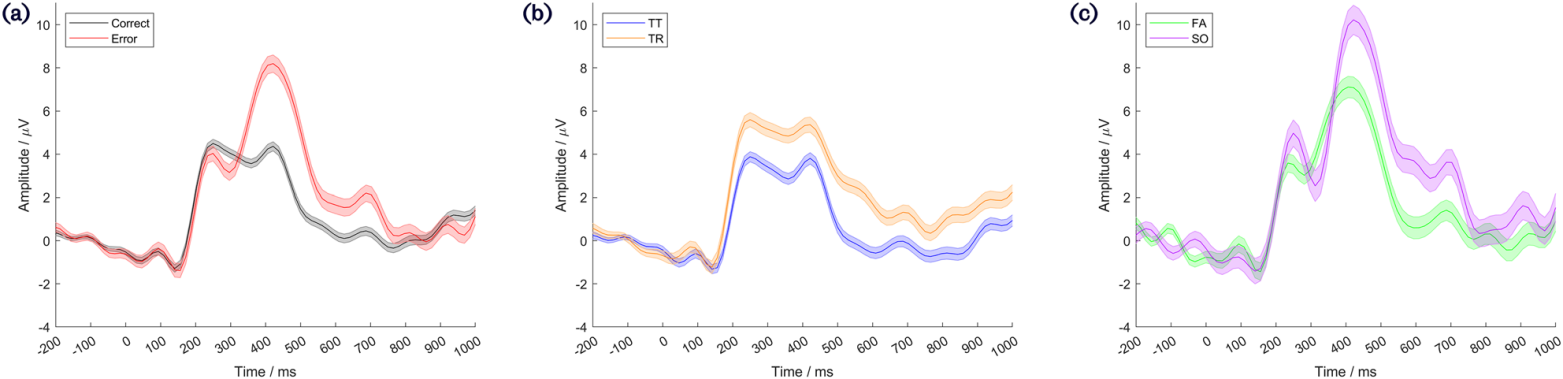
Grand average time domain EEG data generated as subjects observed virtual robot movements. (**a**) shows a comparison of responses to correct movements (black, lower peak) to erroneous movements (red, higher peak). (**b**) shows a comparison of responses to the two correct movements - the TT condition (blue, lower peak), and the TR condition (orange, higher peak). (**c**) shows a comparison of responses to the two erroneous movements - the FA condition (green, lower peak), and the SO condition (purple, higher peak).

Classification of movement actions, — hereafter specified as “movement classification” — was achieved via a 2-stage binary tree. Firstly, EEG trials were classified as responses to either correct (TT and TR condition) or erroneous (FA or SO condition) movements. They were then subclassified as one of the specific conditions. All classifiers used stepwise linear discriminant analysis (SWLDA), which has previously been shown to be successful in classifying event related potentials^30^. The SWLDA algorithm is shown in Supplementary Fig. 1, and has been previously described in detail in our related work, in which it has proven successful with small and imbalanced classes due to its ability to select a small, highly discriminative feature set^28^. The inputs were time domain EEG samples from 200ms to 700ms relative to the robot’s action, from the 8 electrodes. Data were bandpass filtered, downsampled to 64Hz, and baseline corrected to a period from −200ms to 0ms, relative to the robot’s action. For the first stage of classification (error vs correct), a passband of 1 to 10Hz was used, as low frequencies have generally proven fruitful in error detection studies^12^. For the second stage (subclassification), a passband of 1 to 32Hz was used, as the inclusion of information at higher frequencies has previously proven successful in subclassifying similar navigation observations^27^. These data preprocessing steps are visualised in Supplementary Fig. 2. Grand average time domain data showing a comparison of EEG responses to correct vs erroneous movements, as well as each of the subclassifications, are shown in Fig. 2.

Additionally, binary classification of target identification actions — hereafter referred to as “TI classification” — was carried out for each participant, in attempt to gather further information about whether the identified location was the correct target. For all EEG trials in which the robot identified its current location as the target, a classification model was built to classify correct target identifications — hereafter referred to as the “CTI” condition, against false ones — the “FTI” condition. EEG trials were processed in a similar manner to observed movement trials, and were extracted from 200ms to 700ms relative to the yellow box appearing around the robot’s location. Trials for this model were filtered between 1 and 10Hz. Similarly to the movement classification, an SWLDA classifier was used. This TI classification was used as an extra layer of feedback to the robot in the simulated experiments, giving the robot a chance to undo a target identification, if the TI classification output indicated that it was false.

### Navigation strategies

In this study, we assumed that the robot knew the map perfectly - it knew the shortest path between any two locations. As discussed in the *Introduction*, we could imagine that the robot had a satellite navigation system for any route it might need to take and, if a pre-existing map could not be utilised, a preliminary phase of robot path planning could be employed without the need for user input^18–23^. However, the robot did not know which location was the target - this is what the robot needed to learn from the EEG information. Given this assumption, three navigation strategies were used.

#### Proposed Bayesian inference

Kruschke and Liddell describe Bayesian analysis as “reallocation of credibility across possibilities”^31^. In this case, each location on the grid represented a possible target. At the start of each run, each location could be considered to have an equal probability of being the target, but as we gathered more information in the form of EEG responses to the robot’s actions, we could infer that some locations are more credible targets than others. As such, we deemed Bayesian inference to be an appropriate strategy for this scenario of learning to navigate and identify targets.

Let us present the action performed by the robot at time step *t* as $U_t$, where $t\in [1,2,..,l]$ and *l* denotes the last time step in a given run. $U_t$ can be considered as the control state of the model representing that the robot either moved to a neighbouring location, or identified its current location as the target. Subsequently, $S_t$ represents the participant’s interpretation, at time step *t*, of what has occurred as a result action $U_t$. In this study, for movement actions $S_t \in {\mathscr {S}}\!=$[TT, TR, FA, SO], and for target identification actions ${\mathscr {S}}\!=$[CTI, FTI]. This interpretation was not seen directly by the robot, and so can be considered as the hidden state of the model. However, the participant’s interpretation of $U_t$ could be reflected in the recorded EEG signals. Thus, EEG signals resulting from interpretation $S_t$ were classified, providing observation $\varvec{O_t}\!\in \!{\mathscr {O}}$ as the classification output at time step *t*. In this study, each observation was represented as a binary vector with length equal to the number of possible classification outputs. For movement actions, $\varvec{O_t}\!=\!{\mathscr {O}}(1)\!=$[1 0 0 0] represented a classification output of TT, $\varvec{O_t}\!=\!{\mathscr {O}}(2)\!=$[0 1 0 0] represented TR, $\varvec{O_t}\!=\!{\mathscr {O}}(3)\!=$[0 0 1 0] represented FA, and $\varvec{O_t}\!=\!{\mathscr {O}}(4)\!=$[0 0 0 1] represented SO. For target identification actions, $\varvec{O_t}\!=\!{\mathscr {O}}(1)\!=$[1 0] represented CTI, and $\varvec{O_t}\!=\!{\mathscr {O}}(2)\!=$[0 1] represented FTI.

**Figure 3:**
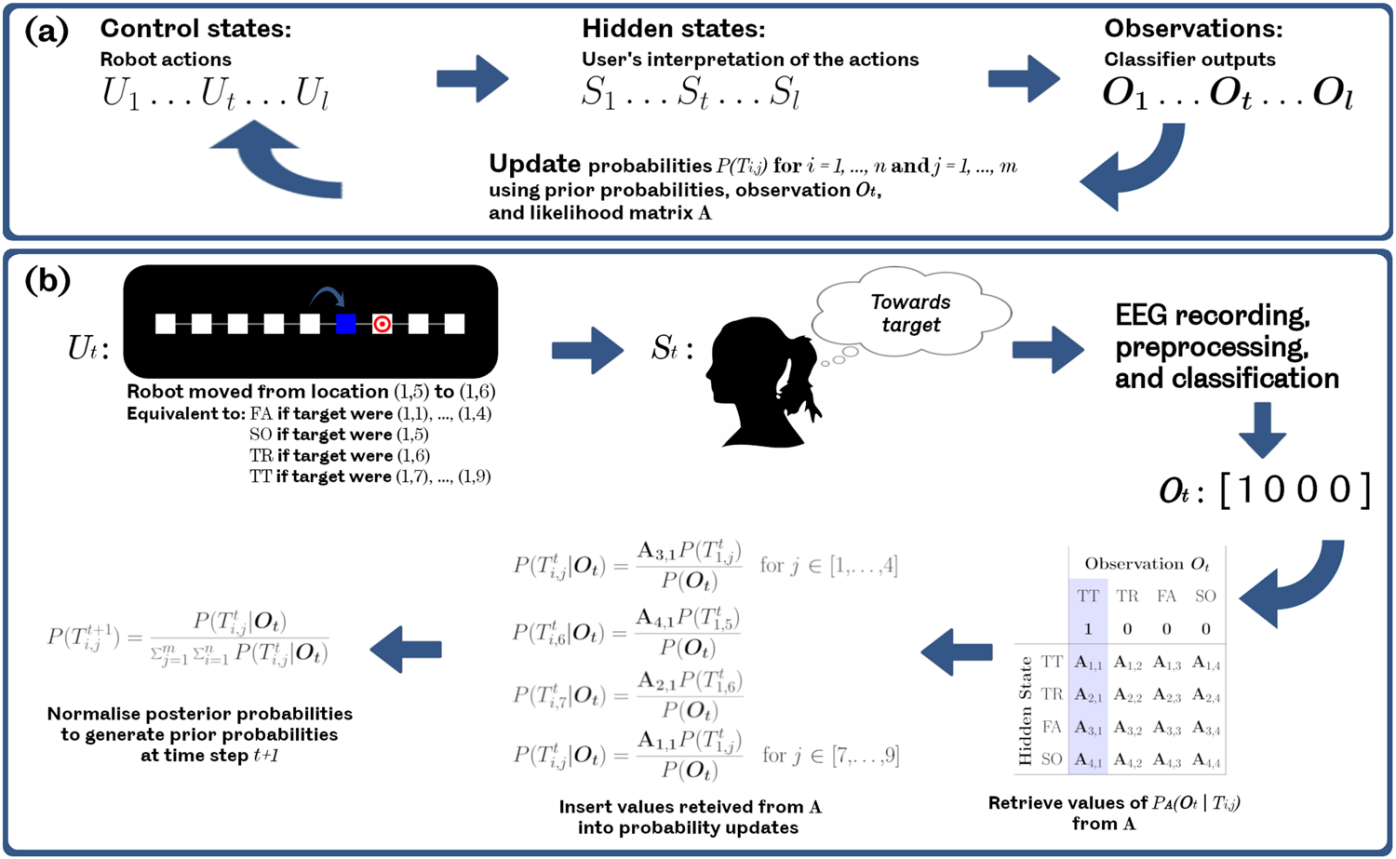
Formulation of the proposed Bayesian inference strategy. (**a**) shows the general case. Robot actions lead to interpretations by the user. These resulted in EEG signals which were given classification outputs. These observations, along with existing target location probabilities and the likelihood matrix ${{\textbf {A}}}$, determined updated target location probabilities. Probabilities informed the next robot actions. (**b**) shows an example of how the Bayesian inference strategy updated the target location probabilities after one movement action, thus influencing future actions. The action $U_t$ was that the robot moved from location (1, 5) to (1, 6). The user saw that this resulted in the robot moving towards the target. The classifier correctly produced the observation representing the TT condition. Values from the appropriate column were extracted from ${{\textbf {A}}}$ and used to update probabilities. If any of locations 1-4 were the target, the action would have represented the FA condition. For location 5, the action would have represented the SO condition. For location 6, the action represented the TR condition. For locations 7 (the actual target), 8, and 9, the action would have represented the TT condition. All probabilities were updated accordingly. The robot would then go on to select the next action, $U_{t+1}$. Depending on the latest classifier output $\varvec{O}_t$ and the updated target location probabilities, action $U_{t+1}$ would either be to select the current location as the target, or to perform another movement action.

Figure 3a shows a representation of the way that each action $U_t$ influenced the participant’s interpretation $S_t$, which lead to a classifier output $\varvec{O_t}$. The probability of each grid location being target was updated as explained below, thus influencing the following action. Depending on the classification output and the updated probabilities, the following action would be either identifying the current location as the target or making the next movement action.

*Defining the next movement action*: Let’s define $P(T^t_{i,j})$ as the probability of a given grid location (*i*, *j*) being the target at time step *t*. Similarly, $P(T^t_c)$ is defined as the probability that the robot’s current location is the target. At the start of each run, these probabilities were equal to $P(T^1_{i,j})= {1}/{(n \times m)}$ for every location of a grid with *n* rows and *m* columns. When a new classification output $\varvec{O_t}$ was observed following a robot action $U_t$, $P(T^{t+1}_{i,j})$ at time step $t+1$ were updated according to Bayes theorem^32^, using (1) and (2);

1 $$\begin{aligned} P(T^{t+1}_{i,j}) = \frac{P(T^t_{i,j} | \varvec{O_t})}{\sum _{j=1}^{m} \sum _{i=1}^{n} P(T^t_{i,j} | \varvec{O_t})}, \end{aligned}$$

2 $$\begin{aligned} P(T^t_{i,j} | \varvec{O_t}) = \frac{P_{\textbf{A}}(\varvec{O_t} | T_{i,j})P(T^t_{i,j})}{P(\varvec{O_t})}. \end{aligned}$$

In (2), $P_{\textbf{A}}(\varvec{O_t} | T_{i,j})$ was calculated using the likelihood matrix ${{\textbf {A}}}$, representing the likelihood of observations given the hidden states. In this study, for the movement actions ${{\textbf {A}}} \subset {\mathbb {R}}^{4\times 4}$, whereas for the target identification actions, ${{\textbf {A}}} \subset {\mathbb {R}}^{2\times 2}$. The elements of ${{\textbf {A}}}$ were calculated as

3 $$\begin{aligned} {{\textbf {A}}}(i,j) = P({\mathscr {O}}(i)|{\mathscr {S}}(j)). \end{aligned}$$

In fact, using ${{\textbf {A}}}$ the robot could estimate the reliability of each classification output. ${{\textbf {A}}}$ was subject-specific. The robot was not allowed to have prior knowledge based on test data. Therefore, for each participant, ${{\textbf {A}}}$ was calculated using leave-one-out cross validation on the classification training EEG data, as this was deemed the closest possible approximation to the model based on all training data. Subsequently, $P_{\textbf{A}}(\varvec{O_t} | T_{i,j})$ was extracted from ${{\textbf {A}}}$, by retrieving the value in the column corresponding to $\varvec{O_t}$, and the row corresponding to what the participant’s perception $S_t$ would have been, if (*i*, *j*) were the target. $P(\varvec{O_t})$ could be also calculated using ${{\textbf {A}}}$, as the sum of the elements in the column corresponding to $\varvec{O_t}$, divided by the sum of all the elements of **A**. In other words, $\varvec{O_t}$ could be calculated using the following equation:

4 $$\begin{aligned} P(\varvec{O_t}) = \frac{\sum _{j=1}^{k}P(\varvec{O_t} |{\mathscr {S}}(j))}{\sum _{i=1}^{k}\sum _{j=1}^{k} P({\mathscr {O}}(i)|{\mathscr {S}}(j))}, \end{aligned}$$

where *k* was the total size of ${\mathscr {S}}$ which is 4 for the movement actions and 2 for the target identifications.

Figure 3b shows an example of how the probabilistic model was updated using the proposed Bayesian inference strategy, after an individual movement action.

After calculating $P(T_{i,j}^{t+1})$ for all the grid locations, the robot identified the location with the highest probability of being the target, and then calculated the shortest path to reach it. The virtual robot then took the first step on this path as $U_{t+1}$. If multiple locations were tied for the highest probability, one was selected at random.

Theoretically, our proposed approach could be extended, such that $\varvec{O_t}$ could represent the likelihood of each class, rather than being binary. For example, if $\varvec{O_t}$ were to equal [0.75 0.25 0 0], i.e. a 75% probability of class 1 and a 25% probability of class 2, then instead of selecting the values of $P_{\textbf{A}}(\varvec{O_t} | T_{i,j})$ being extracted from a single column in ${{\textbf {A}}}$, the values would be calculated as a weighted average of 0.75 $\times$ column 1 of ${{\textbf {A}}}$ and 0.25 $\times$ column 2 of ${{\textbf {A}}}$.

*Identifying the current location as target*: In order to determine whether the robot’s current location *c* was the target, after the target location probabilities had been updated following each movement action, algorithm 1 was run. *stringency* is a predefined variable to determine the level of certainty the system required before identifying the robot’s current location as the target. In this study, values between 0.1 and 0.9 were used.
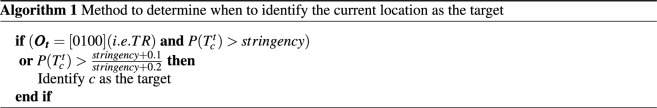


According to algorithm 1, there were two scenarios in which the robot could select a target:

- The current movement classification output $\varvec{O_t}$ represented TR and a lower probability threshold was met.
- The current movement classification output $\varvec{O_t}$ did not represent TR but a higher probability threshold was met.

At the lowest *stringency* value of 0.1, this meant a movement classification output of TR resulted in the target being identified as long as the probabilistic model believed there to be more than a 10% chance of the current location *c* being the target. Alternatively, without a movement classification output of TR, the current location *c* would still be identified if it was considered more than twice as likely to be the target as all other loci combined. As *stringency* increased, the required probabilities — and thus the strength of evidence that had to be accumulated in support of a given location being the target, either with or without a classification output of TR — increased. The proposed Bayesian inference strategy allowed the possibility of deselecting targets. Following a target identification action, if the TI classification output was FTI, the target was deselected. Thereafter, all probabilities $P(T^t_{i,j})$ were updated using (1) and (2), with the likelihood matrix of target identification actions, ${{\textbf {A}}} \subset {\mathbb {R}}^{2\times 2}$. The run continued until a target identification action was followed by a TI classification output of CTI.

Examples of paths taken by the robot when following the Bayesian inference strategy, in small and large grids, and with stringency values of 0.1 and 0.9, are shown in Supplementary Fig. 3.

#### React

The next strategy, *React*, used EEG classifications to inform immediate moves, but did not involve any broader learning. This strategy effectively put 100% trust in the most recent EEG classification output.

If the classifier had not identified the robot’s current location *c* as the target, the robot would move to a neighbouring location, selected at random from a list of eligible neighbours of the robot’s current location. For the first action of each run, all neighbours were eligible. After action $U_t$, a pre-recorded EEG trial from the appropriate class was processed, and classification output $\varvec{O_t}$ was produced by the trained model. If $\varvec{O_t}$ suggested that the action was the TT condition (moving towards the target but not reaching it), then the robot’s previous position would be ruled out from the list of eligible neighbours. Locations were only ruled out for a single action, as the most recent classification output superseded previous ones. If $\varvec{O_t}$ suggested that the action was an erroneous movement (FA or SO condition), the list of eligible neighbours would be reduced to only the previous position, and therefore action $U_{t+1}$ would be to move back to the robot’s previous location, undoing the error. Finally, if $\varvec{O_t}$ suggested the target had been reached (TR condition), then the robot’s position *c* would be identified as the target location.

When the yellow box was drawn to identify the target, TI classification was performed in order to classify the identification as either correct (CTI) or false (FTI). If the classification output was FTI, the identification would be undone, and the run would continue. The run would end when a movement action received a TR movement classification output, then the yellow box was drawn around the robot’s location, and the TI classification output was CTI.

#### Random

As a performance baseline, a random strategy was implemented. For each action, with probability of ${1}/{(n \times m)}$ (i.e. 1/9 on the 9$\times$1 grid, 1/400 on the 20$\times$20 grid), the robot’s current position *c* would be identified as the target location. Otherwise, a neighbouring position would be selected at random, and the robot would move there. The process would repeat until the target was identified, at which point the run would end. While this strategy did not require EEG input, 1000 runs were still simulated for each participant.

### Assessing the effect of detailed EEG information

In this study, our proposed Bayesian inference strategy made use of detailed EEG information. We implemented 4-way classification of movement actions, as opposed to the state-of-the-art approach using binary classification. Furthermore, we included the classification of target identification actions.

In order to investigate the effect of TI classification, we compared our proposed Bayesian inference strategy to an equivalent system which had the TI classification feature switched off. In this case, each run ended as soon as the first target identification action was performed.

We also compared the proposed system to one using state-of-the-art, binary error vs correct movement classification. The binary system was equivalent to the Bayesian inference strategy, with two key changes. Firstly, the likelihood matrix ${{\textbf {A}}}$ for movement classifications was reduced from a 4$\times$4 matrix (TT, TR, FA, and SO) to a 2$\times$2 matrix (correct and erroneous movements). Secondly, as specific TR classification outputs were no longer available, the target would simply be identified if $P(T^t_c)\!>\!\frac{stringency+0.1}{stringency+0.2}$. For both of these alternative systems, 1000 simulations were run for each participant, and for each of the small and large grids, with *stringency* values ranging from 0.1 to 0.9.

## Results

### Evaluation of navigation strategies in small and large grids

Navigation strategies were compared using two metrics, namely *PTCI* and *MNS*. To assess accuracy, we calculate the percentage of targets correctly identified (*PTCI*). Higher *PTCI* represents greater accuracy. To assess speed, we calculate the mean normalised number of steps (*MNS*) taken to achieve correct target identifications. Steps are normalised to represent the efficiency of the path taken, accounting for the fact that the virtual robot starts each run a variable number of steps away from the target.

#### Percentage of targets correctly identified (PTCI)

**Figure 4:**
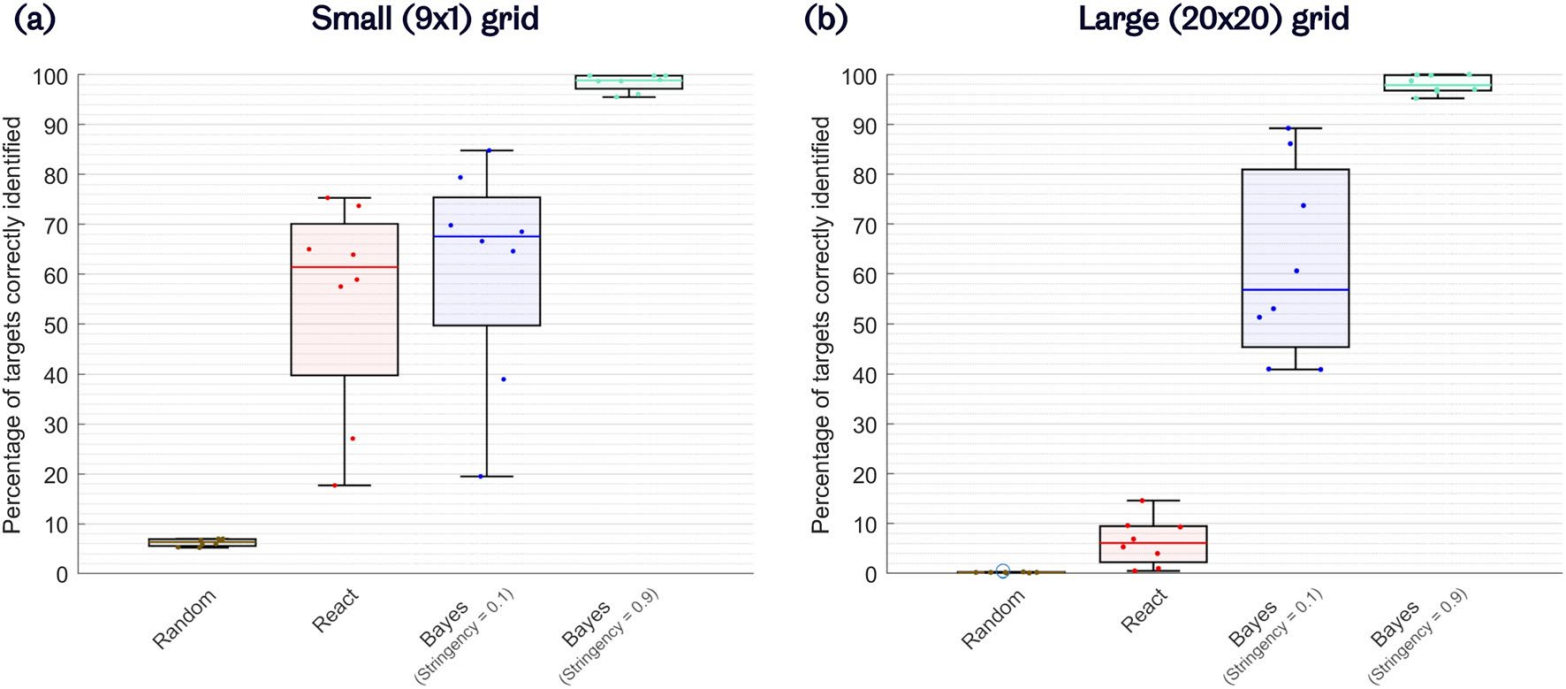
PTCI (percentage of targets correctly identified) performance of each navigation strategy on (**a**) the small (9$\times$1) grid, and (**b**) the large grid, across the 8 participants. Higher PTCI represents greater accuracy.

A comparison of the strategies’ *PTCI* on the small, 9$\times$1 grid across the 8 participants is shown in Fig. 4a. The *Random* strategy achieved a mean *PTCI* of only 6.2% (s.d. 0.7%). The PTCI increased to a mean of 54.9% (s.d. 21.2%) for the *React* strategy, and increased further to a mean of 61.5% (s.d. 21.7%) for the proposed Bayesian inference strategy with a *stringency* value of 0.1. At the highest *stringency* level of 0.9, a mean *PTCI* of 98.4% (s.d. 1.7%) was achieved.

In the large, 20$\times$20 grid, the *PTCI* for the *Random* and *React* strategies were effectively negligible, at just 0.2% (s.d. 0.1%) and 6.4% (s.d. 4.7%), respectively, as shown in Fig. 4b. Conversely, our proposed Bayesian inference strategy retained strikingly high *PTCI* when scaling to the large grid. At a *stringency* level of 0.1, a mean *PTCI* of 62.0% (s.d. 19.0%) was achieved in the large grid. At a *stringency* level of 0.9, very nearly all targets were correctly identified: the mean *PTCI* was 98.03% (s.d. 1.8%). Indeed, for one participant at this highest *stringency* setting, 100% of the 1000 targets in the large grid were identified correctly.

A 2 (grids: small and large) $\times$ 3 (navigation strategies: *Random*, *React*, and Bayesian inference with a *stringency* level of 0.1) repeated measures ANOVA was performed on the *PTCI* results. The statistical results revealed significant main effects of grid size ($p = 0.001$) and navigation strategy ($p < 0.001$) on the *PTCI*. Moreover, a significant interaction between the grids and the navigation strategies were observed, regarding *PTCI* ($p < 0.001$).

Post hoc analysis revealed that, after Bonferroni correction, the proposed Bayesian inference strategy significantly outperformed both *Random* ($p < 0.001$) and *React* ($p < 0.001$) strategies in terms of *PTCI*. The *React* strategy also significantly outperformed the random strategy ($p = 0.001$). Interestingly, when comparing the proposed Bayesian inference strategy with the *React* strategy, a significant interaction was revealed between grid size and navigation strategy ($p < 0.001$). This shows that the *React* strategy’s *PTCI* is much more negatively affected by the change from small to large grid than the proposed Bayesian inference strategy. In fact, the proposed Bayesian inference strategy was quite robust to the increase in grid size. Therefore, the proposed Bayesian inference strategy not only provided the best target recognition accuracy of all the tested strategies, but was also shown to be a scalable approach.

#### Mean normalised steps (MNS)

**Figure 5:**
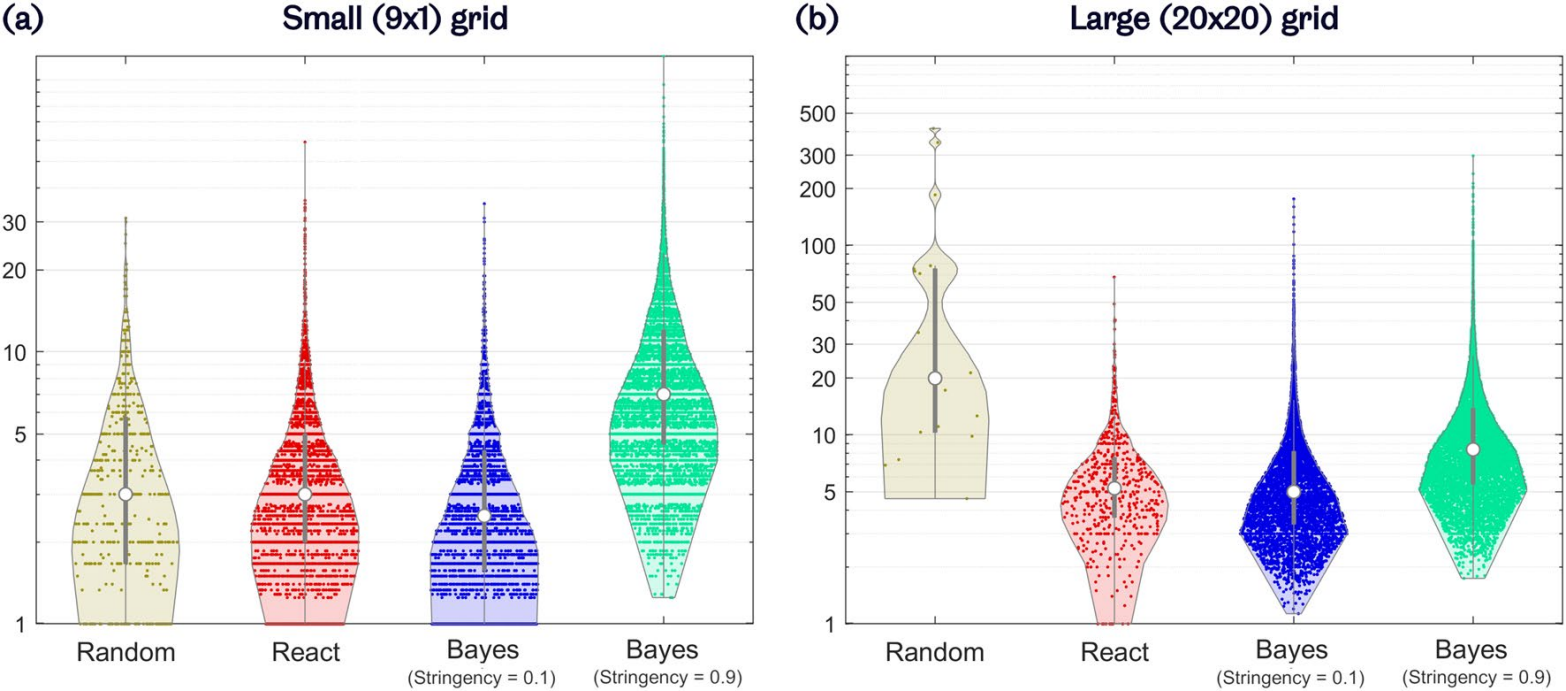
MNS (mean normalised steps) performance of navigation strategies on (**a**) a small (9$\times$1) grid and (**b**) a large (20$\times$20) grid. Violin plots show the smoothed distribution of normalised number of steps to correctly identify targets. *React* and Bayesian strategy distributions are based on data from all participants, combined. Lower MNS represents greater speed. The y-axis is plotted on a logarithmic scale. Median values identified by white dots.

Violin plots comparing the strategies’ *MNS* on the 9$\times$1 grid are shown in Fig. 5a. The distributions for these plots are based on all runs in which the target was correctly identified, from all participants, combined. Using the *Random* strategy, the *MNS* was 4.4 (s.d. 0.4). With the *React* strategy, the *MNS*, calculated across the averages for each participant, was reduced to 3.9 (s.d. 1.5). This reduced further for the Bayesian inference strategy with *stringency* level of 0.1, to 3.3 (s.d. 0.9).

An increase in *MNS* (i.e. decrease in speed) was observed when expanding to the large grid, as shown in Fig. 5b. This is to be expected as there is a change from 1 dimension to 2. With the *Random* strategy, the increase in *MNS* was very large, to a mean of 72.4 (s.d. 76.4). With both the *React* and Bayesian inference (*stringency* = 0.1) strategies, the change in *MNS* was much smaller, increasing to 6.4 (s.d. 3.6) and 7.8 (s.d. 3.7), respectively.

A 2 (grids: small and large) $\times$ 3 (navigation strategies: *Random*, *React*, and Bayesian inference with a *stringency* level of 0.1) repeated measures ANOVA was performed on the *MNS* results. Greenhouse-Geisser correction was applied to this ANOVA, as the sphericity assumption did not hold for *MNS* data. Again, significant main effects of both grid size ($p = 0.031$) and navigation strategy ($p = 0.040$) were reported, as well as a significant interaction between grid size and navigation strategy ($p = 0.045$). This further substantiates the point that, while the *Random* strategy’s *MNS* was severely affected by an increase in grid size, this change had a significantly smaller effect on both the *React* strategy and the proposed Bayesian inference strategy.

Post hoc analysis, after Bonferroni correction, did not show any significant pairwise differences between navigation strategies in terms of *MNS*.

### The speed-accuracy trade-off

By changing the *stringency* setting, we can choose to put more focus on either the speed (*MNS*) or the accuracy (*PTCI*) of the system. There is a trade-off - generally, increasing performance in one of these metrics means decreasing performance in the other.

**Figure 6:**
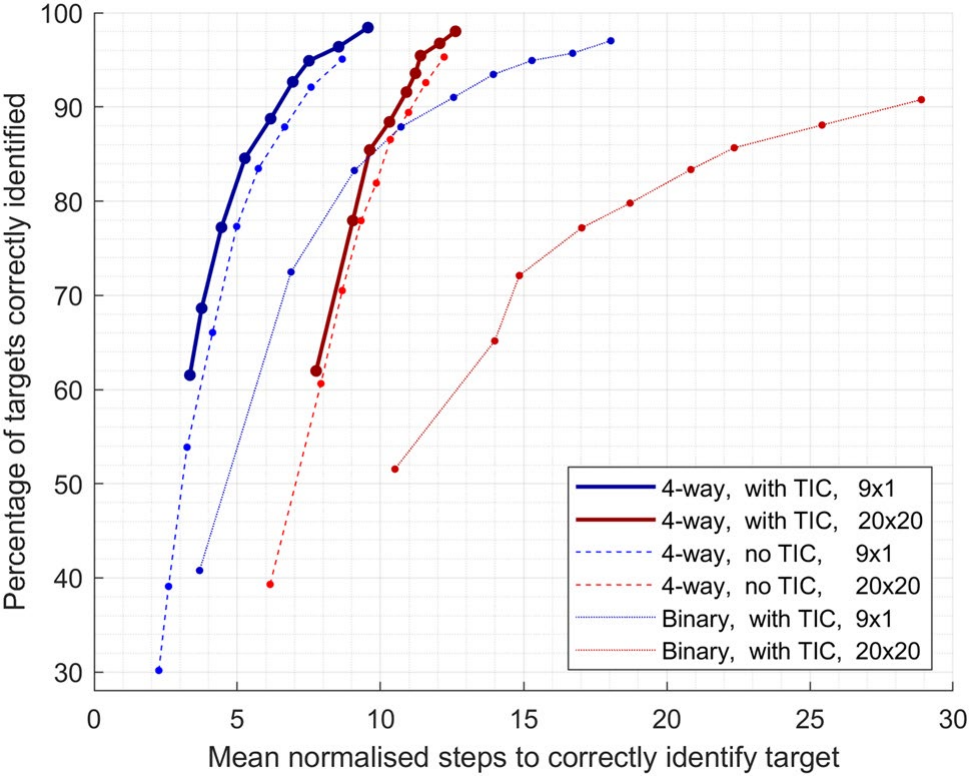
Speed and accuracy trade-off. MNS and PTCI results at various *stringency* values, from 0.1 (lowest point of each line on both axes) to 0.9 (highest point of each line on both axes). Lower MNS represents greater speed. Higher PTCI represents greater accuracy. Bold, solid lines represent data generated using the proposed Bayesian inference system (with 4-way movement classification, and TI classification, here abbreviated to “TIC”). Dashed lines represent data generated using a system without target identification classification. Dotted lines represent data generated using a system with binary movement classification (i.e. correct versus error). Blue lines represent data from the small, 9$\times$1 grid. Red lines represent data from the large, 20$\times$20 grid. All lines show average results across all participants.

Using the Bayesian inference strategy, we calculated *PTCI* and *MNS*, in both small and large grids, with *stringency* values in increments of 0.1 between 0.1 and 0.9. The results of these calculations are shown in Table 1. The trade-off is demonstrated by the high correlation coefficients between *PTCI* and *MNS*: $r = 0.94$ in the 9$\times$1 grid and $r = 0.95$ in the 20$\times$20 grid ($p = 1.6\times 10^{-4}$ and $p = 6.2\times 10^{-5}$, respectively). A visualisation of this trade-off is shown, for both the small and large grids, in Fig. 6 (solid lines).

**Table 1:** Percentage of targets correctly identified (PTCI), and the mean normalised number of steps taken in order to identify them (MNS), using the Bayesian inference strategy, with various values of the stringency variable, on both the small and large grids.

Stringency	9 $\times$ 1 grid		20 $\times$ 20 grid	
	PTCI	MNS	PTCI	MNS
0.1	61.5% ± 21.7%	3.3 ± 0.9	62.0% ± 19.1%	7.8 ± 3.7
0.2	68.6% ± 17.5%	3.8 ± 0.9	77.9% ± 13.8%	9.0 ± 4.9
0.3	77.2% ± 13.2%	4.4 ± 0.9	85.4% ± 10.3%	9.6 ± 5.1
0.4	84.6% ± 10.1%	5.3 ± 1.1	88.4% ± 8.4%	10.3 ± 6.1
0.5	88.8% ± 8.0%	6.2 ± 1.3	91.6% ± 6.3%	10.9 ± 6.1
0.6	92.7% ± 5.2%	6.9 ± 1.7	93.6% ± 5.1%	11.2 ± 6.2
0.7	94.9% ± 3.8%	7.5 ± 1.8	95.5% ± 3.9%	11.3 ± 5.3
0.8	96.4% ± 3.1%	8.5 ± 2.2	96.8% ± 2.8%	12.1 ± 6.2
0.9	98.4% ± 1.7%	9.6 ± 2.8	98.0% ± 1.8%	12.6 ± 6.4

Results shown represent mean ± standard deviation across all participants.

On average, it was possible to achieve a *PTCI* of over 90% with a *stringency* setting of 0.6 in the small grid, and 0.5 in the large grid. These settings afforded a mid-range *MNS*, at an average of 6.9 and 10.9 in the small and large grids, respectively. For applications where the very highest *PTCI* (i.e. the highest accuracy) is required, we can achieve this in either the small or large grid by setting the *stringency* to 0.9. Of course, this does require more steps in order to attain the higher threshold of confidence that the target has been reached. With this very high *stringency* value, the *MNS* increased to an average of 9.6 and 12.6 in the small and large grids, respectively. The reward is near-perfect accuracy: In both grids, an average *PTCI* of over 98% was achieved, with *PTCI* of over 95% for every single participant.

There was also a high negative correlation between *stringency* and the standard deviation from the mean *PTCI* ($r = -0.97$, $p = 1.4\times 10^{-5}$ in the small grid; $r = -0.95$, $p = 6.2\times 10^{-5}$ in the large grid). Conversely, there was a very high positive correlation between *stringency* and the standard deviation from the mean *MNS* ($r = 0.95$, $p = 1.1\times 10^{-4}$ in the small grid; $r = 0.78$, $p = 0.01$ in the large grid). In other words, as we require more evidence in order to identify each target, *PTCI* gets more consistent across participants. Meanwhile, *MNS* is more consistent across participants when the *stringency* is lower, and becomes more varied as *stringency* increases. Therefore, if an application were to require consistency across users in either speed or accuracy, these features can also be controlled by tuning the *stringency* parameter.

### The effect of classifying the target identification action

As discussed in the Methods section, ‘Assessing the effect of detailed EEG information’, simulations were also run with the TI classification feature switched off. The results of these simulations are shown as the dashed lines in Fig. 6. Existing state-of-the-art approaches generally use error-vs-correct classification of movements, but do not include any classification of target identification actions. Therefore, these simulations allow us to compare our more detailed system to state-of-the-art methodology which does not include this additional classification.

Paired Wilcoxon signed-rank tests found the *PTCI* achieved with TI classification to be significantly higher ($p < 0.05$) than that achieved without TI classification at every *stringency* level in both the small grid and large grids. As we might expect, targets are generally identified slightly faster on average without TI classification, as no extra steps can be taken after an initial target identification action. This difference in *MNS* was found to be significant ($p < 0.05$) at all but the highest *stringency* level in the small grid. However, in the large grid, the difference in *MNS* was only found to be significant at 3 of the 9 *stringency* levels.

The largest effect size of TI classification occurs at the lowest *stringency* levels. In these cases, we can reasonably expect more false target identifications to occur, as less evidence needs to be accumulated in order to perform a target identification action. In the $9\times 1$ grid, *PTCI* of 61.5% (s.d. 21.7%) was achieved with TI classification, as opposed to just 30.2% (s.d. 11.4%) without TI classification. In the $20\times 20$ grid, 62.0% (s.d. 19.1%) *PTCI* was achieved with TI classification, compared to 39.3% (s.d. 15.4%) without. This indicates that TI classification strongly improves the robustness of the system.

### Comparison of 4-way vs binary classification

We compared the efficiency of the system using the proposed 4-way movement classification against one using the existing state-of-the-art, binary error vs correct movement classification. The results of the simulations using binary movement classification (as discussed in the Methods section, ‘Assessing the effect of detailed EEG information’) are shown as the dotted lines in Fig. 6.

Paired Wilcoxon signed-rank tests for each *stringency* level of each grid revealed that only in the small grid with *stringency* of 0.1 were the *PTCIs* significantly different ($p < 0.01$). In this case, 4-way classification achieved a higher *PTCI*.

The more striking effect of 4-way classification is on the speed of the system. As can be seen in Fig. 6, 4-way movement classification allows convergence on correct targets in fewer steps, on average, than binary movement classification. This difference in *MNS* was statistically significant ($p < 0.05$) in 8 out of 9 *stringency* levels for each of the small and large grids.

It is also noteworthy that we observed smaller standard deviations in the *MNS* with 4-way movement classification than with binary classification. Further paired Wilcoxon signed-rank tests, comparing standard deviations of *MNS* at different stringency levels between 4-way and binary movement classification, found the 4-way approach to have significantly smaller standard deviations in both the small (4-way range: 0.9 to 2.8, binary range: 1.5 to 13.1, $p < 0.01$) and large (4-way range: 3.6 to 6.4, binary range: 7.5 to 36.3, $p < 0.01$) grids.

Therefore, when using 4-way movement classification, we have seen significant improvements in speed, as well as significantly increased consistency of speed between participants.

## Discussion and conclusion

This study has presented a scalable approach that makes it possible to use reactive EEG not only to navigate towards target locations, but also to accurately identify the correct targets once they have been reached. All strategies using EEG correctly identified several times more targets than the *Random* approach. The EEG-based strategies were also capable of identifying these targets in fewer steps than the *Random* approach. Our proposed Bayesian inference strategy, which iteratively updated a probabilistic model to learn the most likely target location, proved to be the most efficient of the navigation strategies tested in this study. The Bayesian strategy also maintained very high accuracy — here defined as the percentage of targets correctly identified (*PTCI*) — when expanded to a large grid, in which any of 400 spaces could be the target.

For the first time, we have shown a system that uses 4-way classification of EEG responses to robotic movements as feedback for learning-based navigation. This provided contextualised feedback to the robot, including specific information regarding when the target location had been reached. Our results demonstrate that such detailed classification can lead to a more efficient semi-autonomous robot navigation than can be achieved with binary, error vs correct EEG classification alone. We therefore recommend the use of detailed EEG classification where possible in reinforcement learning-based BCIs.

Additionally, binary classification was performed on target identification actions, classifying them as either correct or false. Reliable classification of target identification actions provides the system with an increased level of robustness, due to ability to undo false identifications.

Our proposed Bayesian inference strategy includes an adjustable parameter: *stringency*. By tuning this parameter, we can make the system require a higher or lower threshold of certainty to be met before identifying a target. As such, we can amend the strategy to focus on reaching targets more quickly, or identifying them with greater accuracy, making this approach applicable in a wide range of scenarios. In the fastest case, targets were correctly identified after a mean of 3.3 normalised steps, indicating that a small number of errors can be enough to teach the robot. Interestingly, in the most accurate case, an average of more than 98% of targets were identified correctly.

It is clear that the novel Bayesian inference strategy can be considered preferable to the state-of-the-art. At a stringency level of 0.1, this strategy performed at comperable speed to the “React” strategy, but correctly identified significantly more targets, especially in a 2-dimensional space. Within the Bayesian inference strategy, the precise stringency setting that would provide optimal performance would depend on the task in question, and the user’s preference, regarding whether speed or accuracy were more important. This study provides an exciting step forward by creating the possibility of near-perfect accuracy. However, we have investigated the speed-accuracy trade-off because the number of steps required to reach this accuracy may still be considered impractical or frustrating for some users. In future, we suggest that this work could be extended into a long-term system, in which users’ most commonly preferred targets are learned, allowing initial prior knowledge to be utilised from the beginning of each run. We postulate that this would be one potential way to increase the speed at which highly accurate navigation is achieved.

The methods presented in this study should not be considered the only feasible solutions for semi-autonomous, reactive EEG-based tasks. Indeed, further exploration of other learning methodologies would be welcome additions to the field. For example, one active area of interest in machine learning is inverse reinforcement learning (IRL). An alternative approach to this task could be for a user to initially navigate using active EEG-based control, and for the machine to learn the user’s preferences via deep IRL techniques such as deep-Q networks^33^, implicit quantile networks^34^, or rainbow^35^. These are only a few examples, and this developing field would benefit from broad exploration. However, through the methods utilised in this study, we have shown that (a) the use of increasingly detailed information from reactive EEG, and (b) the intelligent application of contextualised probabilistic modelling improves the performance of semi-autonomous systems.

We have shown the capability for a robot to learn user intentions from reactive EEG signals in a more efficient and accurate manner than ever before. These signals are obtained while users simply observe the robot’s actions, providing a form of implicit communication, and so reducing the mental workload of the user. Such capability could be extremely useful for assistive robotics. Importantly, our system also represents the first demonstration that such semi-autonomous BCIs, guided by reactive brain signals, can be scalable. This shows the potential for these systems to be utilised in large-scale applications. We have presented robotic navigation as an exemplar in this study. However, the principles and techniques could theoretically be generalised and applied to any scenario in which BCI users select a number of preferences at varying rates. Therefore, this study represents important steps forwards for semi-autonomous BCIs, and for efficient, user-friendly human-machine interaction.

### Supplementary Information

Supplementary Information.

## Data Availability

The data used in this study are available for download, under a CC BY-NC-SA 4.0 licence, from: 10.15131/shef.data.23556273.
