# Supplementary material for: Bayesian learning from multi-way EEG feedback for robot navigation and target identification
Source: Sci Rep. 2023 Oct 7;13:16925. doi: 10.1038/s41598-023-44077-8 (PMC10560278; doi:10.1038/s41598-023-44077-8)
Supplement: Supplementary file 1 — Supplementary Information. [file 41598_2023_44077_MOESM1_ESM.pdf]

Supplementary Information for, “Bayesian Learning from Multi-Way EEG Feedback for Robot Navigation and Target Identification”

Christopher Wirth, Jake Toth, and Mahnaz Arvaneh

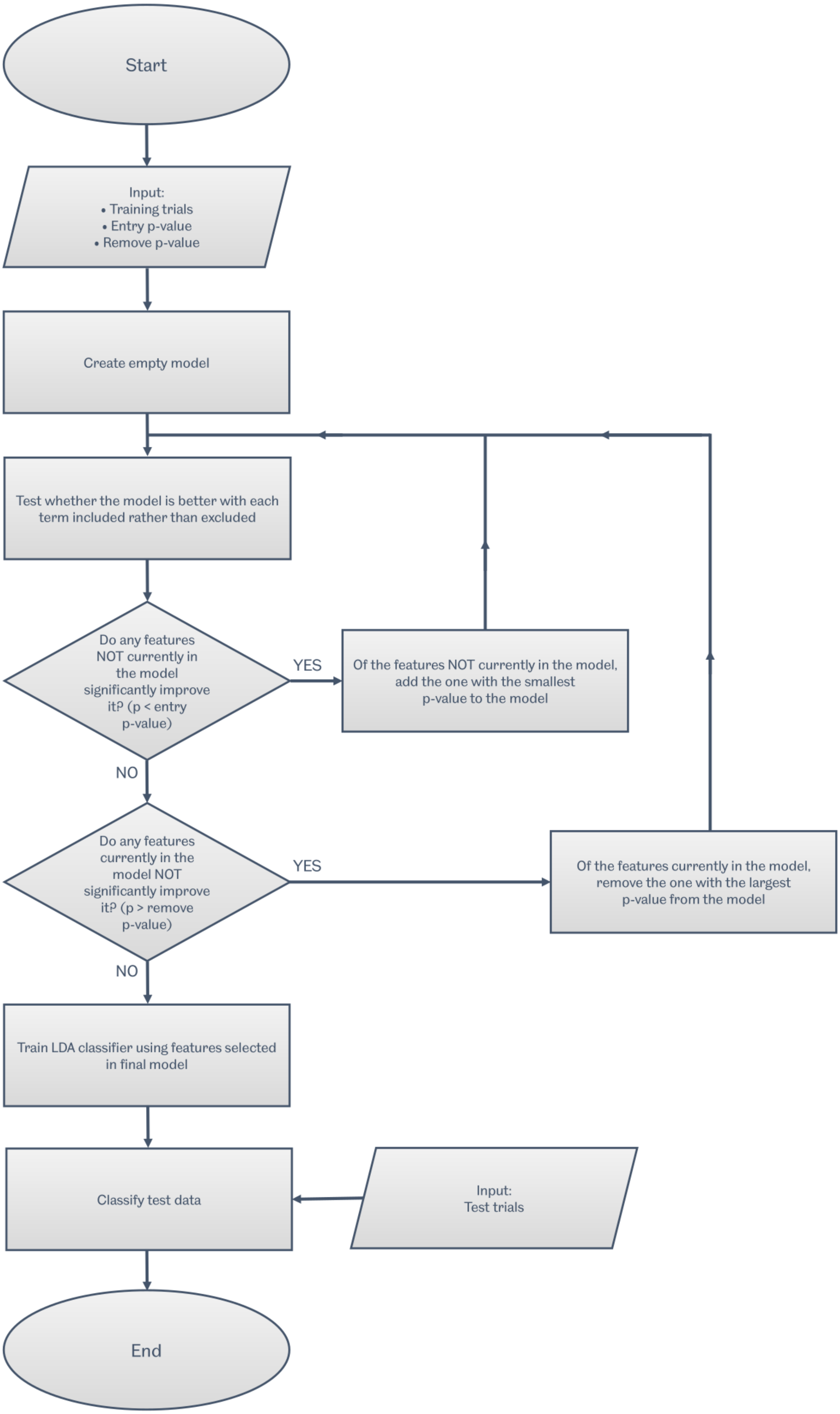


**Supplementary Figure 1.** The Stepwise Linear Discriminant Analysis algorithm. The entry p-value used for this study was 0.025. The remove p-value used for this study was 0.075.

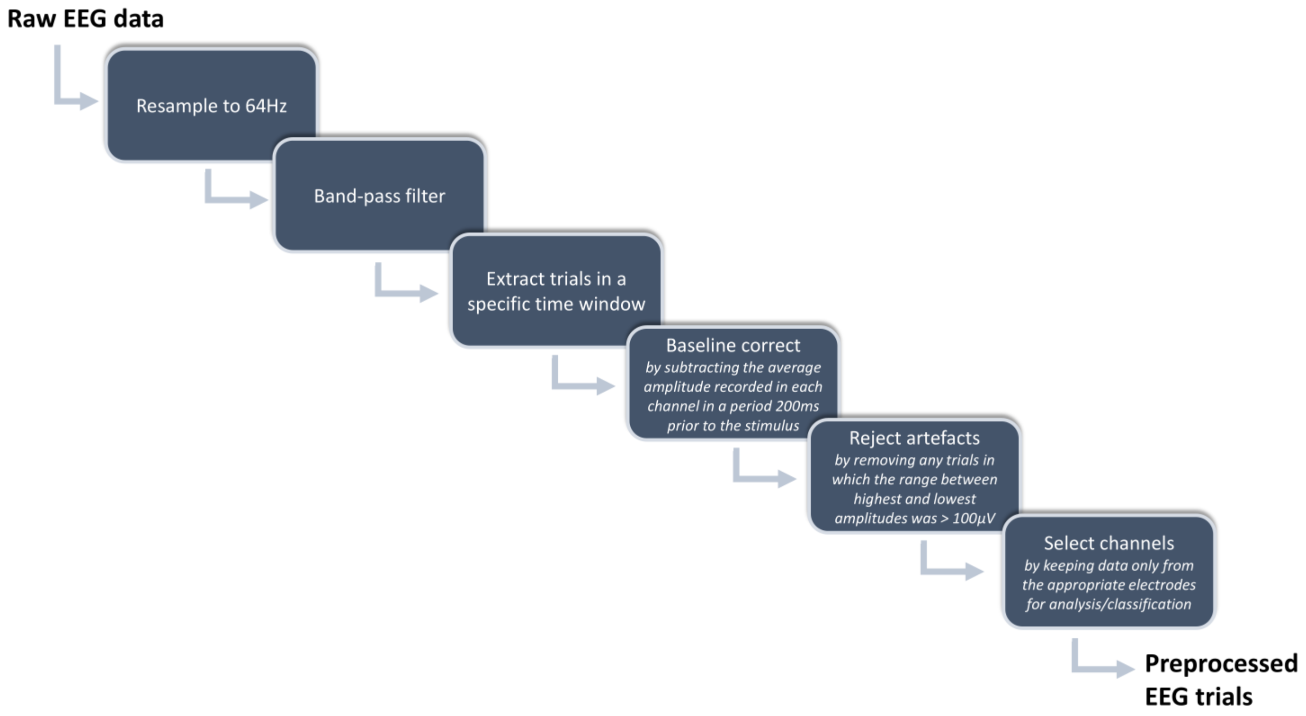


**Supplementary Figure 2.** EEG data preprocessing. Band pass filter high and low frequencies, start and end time points of the time windows, and specific channels used, are all variable depending on the requirements of the analysis. For data visualisation, artefact rejection occurred if the threshold were surpassed in any channel. For classification, artefact rejection occurred only if the threshold were surpassed in a channel that was intended to be used for classification.

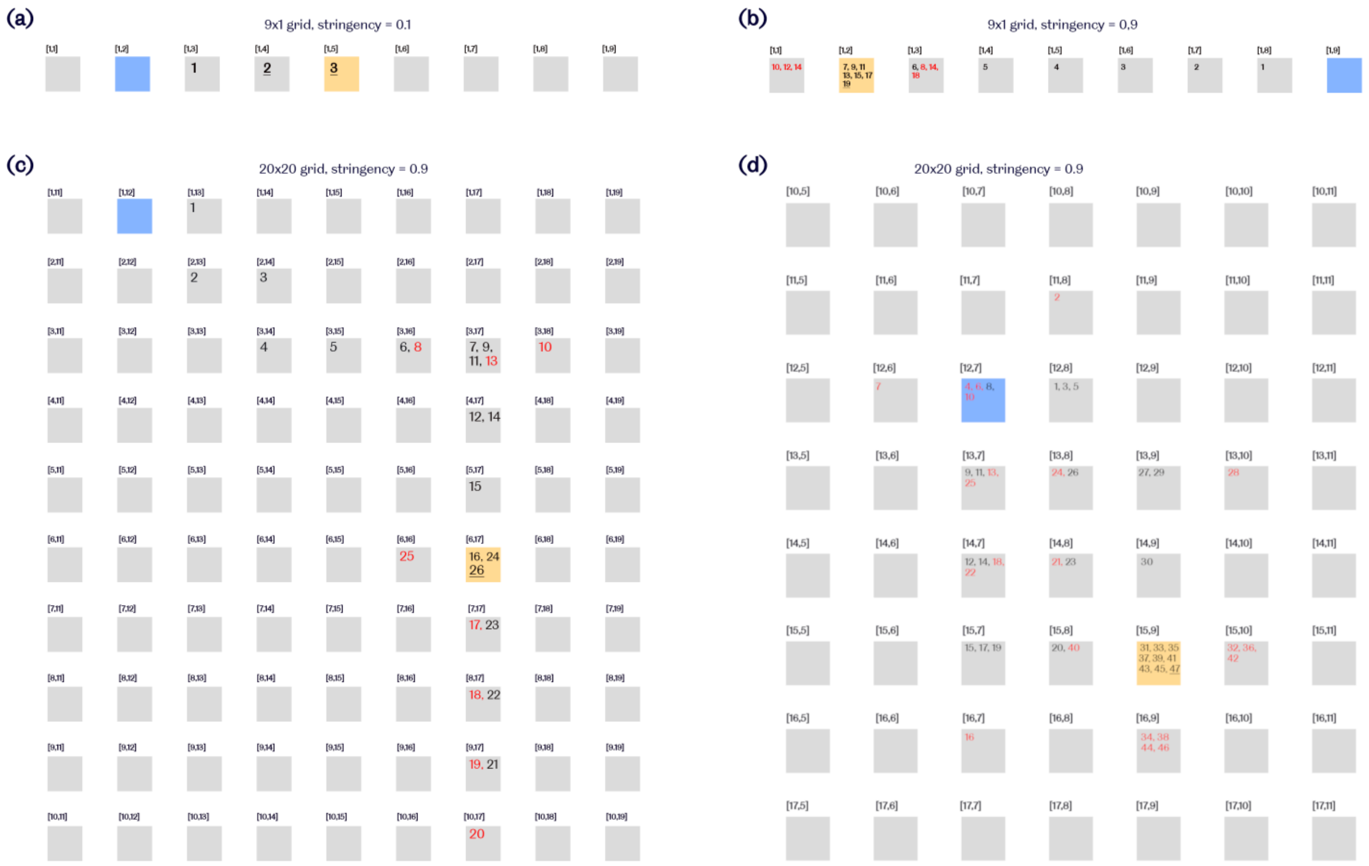


**Supplementary Figure 3.** Example paths taken by the Bayesian inference strategy. Target loci are highlighted in yellow. The cursor's starting location in each example is highlighted in blue. Coordinates are shown above each location. Numbers inside boxes represent the order of the virtual robot's movement actions, e.g. the first move in each example was from the blue square to the square containing the number 1. Some squares contain multiple numbers, separated by commas, as it was possible for a location to be visited multiple times during a run. Action numbers in black text represent correct actions (TR if written in the yellow target square, TT otherwise). Action numbers in red text represent erroneous actions (SOT if following a number in the yellow target box, MFFT otherwise). Underlined numbers indicate that the robot identified its location as the target following the move. (a) shows an example in the 9x1 grid, with stringency 0.1, using EEG data from participant 3. In this example, the robot erroneously identified location [1,4] as the target after move 2. However, this target identification was corrected by a False Target Identificatoin classification, and so the run continued until the correct target was identified following move 3. (b) shows an example of the 9x1 grid, with stringecy 0.9, using EEG data from participant 3. (c) shows an example of the 20x20 grid, with stringency 0.1, using EEG data from participant 10. (d) shows an example of the 20x20 grid, with stringency 0.9, using EEG data from participant 10. In both (c) and (d), the full grid is not shown. Loci ranged from [1,1] to [20,20] - those that are outside the limits included in the figure were not navigated to at any point during the runs shown.

Supplementary Table 1

**Number of training samples: movement actions**

| *Participant* | *TT* | *TR* | *MFFT* | *SOT* | *Total* |
|---|---|---|---|---|---|
| *1* | 138 | 73 | 58 | 25 | 294 |
| *3* | 133 | 79 | 33 | 21 | 266 |
| *4* | 139 | 76 | 40 | 19 | 274 |
| *6* | 132 | 75 | 29 | 19 | 255 |
| *7* | 131 | 72 | 33 | 20 | 256 |
| *8* | 133 | 69 | 40 | 18 | 260 |
| *9* | 123 | 65 | 52 | 27 | 267 |
| *10* | 144 | 76 | 40 | 20 | 280 |

Supplementary Table 2

**Number of test samples: movement actions**

| Participant | TT | TR | MFFT | SOT | Total |
|---|---|---|---|---|---|
| 1 | 24 | 13 | 10 | 5 | 52 |
| 3 | 24 | 14 | 6 | 4 | 48 |
| 4 | 24 | 13 | 7 | 3 | 47 |
| 6 | 23 | 13 | 5 | 3 | 44 |
| 7 | 23 | 13 | 6 | 3 | 45 |
| 8 | 23 | 12 | 7 | 3 | 45 |
| 9 | 22 | 11 | 9 | 5 | 47 |
| 10 | 25 | 13 | 7 | 3 | 48 |

Supplementary Table 3

**Number of training samples: target identification actions**

| Participant | CTI | FTI | Total |
|---|---|---|---|
| 1 | 40 | 28 | 68 |
| 3 | 53 | 28 | 81 |
| 4 | 57 | 25 | 82 |
| 6 | 58 | 28 | 86 |
| 7 | 55 | 27 | 82 |
| 8 | 44 | 27 | 71 |
| 9 | 37 | 27 | 64 |
| 10 | 55 | 25 | 80 |

Supplementary Table 4

**Number of test samples: target identification actions**

| Participant | CTI | FTI | Total |
|---|---|---|---|
| 1 | 7 | 5 | 12 |
| 3 | 9 | 5 | 14 |
| 4 | 10 | 5 | 15 |
| 6 | 10 | 5 | 15 |
| 7 | 10 | 5 | 15 |
| 8 | 8 | 5 | 13 |
| 9 | 7 | 5 | 12 |
| 10 | 10 | 4 | 14 |

Supplementary Table 5

<u>Movement action classification</u>

| Participant | TT | TR | MFFT | SOT | Correct vs Error | 4-Way Balanced Accuracy | 4-Way Overall Accuracy |
|---|---|---|---|---|---|---|---|
| 1 | 0.4167 | 0.5385 | 0.5 | 0.2 | 0.7115 | 0.4138 | 0.4423 |
| 3 | 0.7083 | 0.5 | 0.1667 | 0.5 | 0.7708 | 0.46875 | 0.5625 |
| 4 | 0.5833 | 0.6154 | 0.2857 | 0.3333 | 0.766 | 0.454425 | 0.5319 |
| 6 | 0.6087 | 0.3846 | 0.2 | 0.6667 | 0.7955 | 0.465 | 0.5 |
| 7 | 0.5217 | 0.3077 | 0.5 | 1 | 0.7333 | 0.58235 | 0.4889 |
| 8 | 0.4783 | 0.3333 | 0.4286 | 0.3333 | 0.6889 | 0.393375 | 0.4222 |
| 9 | 0.3636 | 0.7273 | 0.3333 | 0.2 | 0.6596 | 0.40605 | 0.4255 |
| 10 | 0.52 | 0.6154 | 0.2857 | 1 | 0.75 | 0.605275 | 0.5417 |
| *Mean* | *0.52508* | *0.50278* | *0.3375* | *0.52916* | *0.73445* | *0.473628125* | *0.489375* |

Supplementary Table 6

<u>Target Identification classification</u>

| Participant | Correct Target Identification | False Target Identification | Balanced Accuracy | Overall Accuracy |
|---|---|---|---|---|
| 1 | 0.8571 | 0.6 | 0.7286 | 0.75 |
| 3 | 0.6667 | 0.8 | 0.7333 | 0.7143 |
| 4 | 0.8 | 0.8 | 0.8 | 0.8 |
| 6 | 0.7 | 0.8 | 0.75 | 0.7333 |
| 7 | 0.8 | 0.8 | 0.8 | 0.8 |
| 8 | 0.875 | 0.6 | 0.7375 | 0.7692 |
| 9 | 0.5714 | 0.8 | 0.6857 | 0.6667 |
| 10 | 0.6 | 0.75 | 0.675 | 0.6429 |
| *Mean* | *0.733775* | *0.74375* | *0.7387625* | *0.73455* |

Supplementary Table 7

**Movement action cross validation:**

**Participant 1**

| | | *Classification* | | | |
|---|---|---|---|---|---|
| | | *TT* | *TR* | *MFFT* | *SOT* |
| *True label* | *TT* | 64 | 33 | 24 | 17 |
| | *TR* | 18 | 32 | 10 | 13 |
| | *MFFT* | 17 | 12 | 20 | 9 |
| | *SOT* | 6 | 4 | 6 | 9 |

Supplementary Table 8

**Target Identification cross validation:**

**Participant 1**

| | | *Classification* | |
|---|---|---|---|
| | | *CTI* | *FTI* |
| *True label* | *CTI* | 17 | 23 |
| | *FTI* | 13 | 15 |

Supplementary Table 9

**Movement action test set:**

**Participant 1**

| | | *Classification* | | | |
|---|---|---|---|---|---|
| | | *TT* | *TR* | *MFFT* | *SOT* |
| *True label* | *TT* | 10 | 6 | 5 | 3 |
| | *TR* | 3 | 7 | 2 | 1 |
| | *MFFT* | 2 | 1 | 5 | 2 |
| | *SOT* | 0 | 1 | 3 | 1 |

Supplementary Table 10

**Target Identification test set:**

**Participant 1**

| | | *Classification* | |
|---|---|---|---|
| | | *CTI* | *FTI* |
| *True label* | *CTI* | 6 | 1 |
| | *FTI* | 2 | 3 |

Supplementary Table 11

**Movement action cross validation:**
**Participant 3**

| | | *Classification* | | | |
|---|---|---|---|---|---|
| | | *TT* | *TR* | *MFFT* | *SOT* |
| *True label* | *TT* | 46 | 54 | 20 | 13 |
| | *TR* | 28 | 34 | 12 | 5 |
| | *MFFT* | 6 | 9 | 9 | 9 |
| | *SOT* | 3 | 5 | 7 | 6 |

Supplementary Table 12

**Target Identification cross validation:**
**Participant 3**

| | | *Classification* | |
|---|---|---|---|
| | | *CTI* | *FTI* |
| *True label* | *CTI* | 38 | 15 |
| | *FTI* | 9 | 19 |

Supplementary Table 13

**Movement action test set:**
**Participant 3**

| | | *Classification* | | | |
|---|---|---|---|---|---|
| | | *TT* | *TR* | *MFFT* | *SOT* |
| *True label* | *TT* | 17 | 4 | 0 | 3 |
| | *TR* | 3 | 7 | 2 | 2 |
| | *MFFT* | 0 | 3 | 1 | 2 |
| | *SOT* | 0 | 1 | 1 | 2 |

Supplementary Table 14

**Target Identification test set:**
**Participant 3**

| | | *Classification* | |
|---|---|---|---|
| | | *CTI* | *FTI* |
| *True label* | *CTI* | 6 | 3 |
| | *FTI* | 1 | 4 |

Supplementary Table 15

**Movement action cross validation:**
**Participant 4**

| | | *Classification* | | | |
|---|---|---|---|---|---|
| | | *TT* | *TR* | *MFFT* | *SOT* |
| *True label* | *TT* | 63 | 26 | 35 | 15 |
| | *TR* | 14 | 43 | 8 | 11 |
| | *MFFT* | 17 | 4 | 11 | 8 |
| | *SOT* | 3 | 3 | 6 | 7 |

Supplementary Table 16

**Target Identification cross validation:**
**Participant 4**

| | | *Classification* | |
|---|---|---|---|
| | | *CTI* | *FTI* |
| *True label* | *CTI* | 43 | 14 |
| | *FTI* | 10 | 15 |

Supplementary Table 17

**Movement action test set:**
**Participant 4**

| | | *Classification* | | | |
|---|---|---|---|---|---|
| | | *TT* | *TR* | *MFFT* | *SOT* |
| *True label* | *TT* | 14 | 6 | 2 | 2 |
| | *TR* | 1 | 8 | 1 | 3 |
| | *MFFT* | 2 | 0 | 2 | 3 |
| | *SOT* | 1 | 0 | 1 | 1 |

Supplementary Table 18

**Target Identification test set:**
**Participant 4**

| | | *Classification* | |
|---|---|---|---|
| | | *CTI* | *FTI* |
| *True label* | *CTI* | 8 | 2 |
| | *FTI* | 1 | 4 |

Supplementary Table 19

**Movement action cross validation:**
**Participant 6**

| | | *Classification* | | | |
|---|---|---|---|---|---|
| | | *TT* | *TR* | *MFFT* | *SOT* |
| *True label* | *TT* | 56 | 45 | 20 | 11 |
| | *TR* | 26 | 32 | 8 | 9 |
| | *MFFT* | 8 | 4 | 14 | 3 |
| | *SOT* | 4 | 1 | 5 | 9 |

Supplementary Table 20

**Target Identification cross validation:**
**Participant 6**

| | | *Classification* | |
|---|---|---|---|
| | | *CTI* | *FTI* |
| *True label* | *CTI* | 45 | 13 |
| | *FTI* | 5 | 23 |

Supplementary Table 21

**Movement action test set:**
**Participant 6**

| | | *Classification* | | | |
|---|---|---|---|---|---|
| | | *TT* | *TR* | *MFFT* | *SOT* |
| *True label* | *TT* | 14 | 6 | 0 | 3 |
| | *TR* | 5 | 5 | 1 | 2 |
| | *MFFT* | 2 | 1 | 1 | 1 |
| | *SOT* | 0 | 0 | 1 | 2 |

Supplementary Table 22

**Target Identification test set:**
**Participant 6**

| | | *Classification* | |
|---|---|---|---|
| | | *CTI* | *FTI* |
| *True label* | *CTI* | 7 | 3 |
| | *FTI* | 1 | 4 |

Supplementary Table 23

**Movement action cross validation:**
**Participant 7**

| | | *Classification* | | | |
|---|---|---|---|---|---|
| | | *TT* | *TR* | *MFFT* | *SOT* |
| *True label* | *TT* | 63 | 30 | 26 | 12 |
| | *TR* | 20 | 23 | 20 | 9 |
| | *MFFT* | 11 | 6 | 9 | 7 |
| | *SOT* | 0 | 7 | 4 | 9 |

Supplementary Table 24

**Target Identification cross validation:**
**Participant 7**

| | | *Classification* | |
|---|---|---|---|
| | | *CTI* | *FTI* |
| *True label* | *CTI* | 39 | 16 |
| | *FTI* | 11 | 16 |

Supplementary Table 25

**Movement action test set:**
**Participant 7**

| | | *Classification* | | | |
|---|---|---|---|---|---|
| | | *TT* | *TR* | *MFFT* | *SOT* |
| *True label* | *TT* | 12 | 6 | 3 | 2 |
| | *TR* | 4 | 4 | 4 | 1 |
| | *MFFT* | 1 | 1 | 3 | 1 |
| | *SOT* | 0 | 0 | 0 | 3 |

Supplementary Table 26

**Target Identification test set:**
**Participant 7**

| | | *Classification* | |
|---|---|---|---|
| | | *CTI* | *FTI* |
| *True label* | *CTI* | 8 | 2 |
| | *FTI* | 1 | 4 |

Supplementary Table 27

**Movement action cross validation:**
**Participant 8**

| | | *Classification* | | | |
|---|---|---|---|---|---|
| | | *TT* | *TR* | *MFFT* | *SOT* |
| *True label* | *TT* | 52 | 39 | 28 | 14 |
| | *TR* | 24 | 28 | 8 | 9 |
| | *MFFT* | 11 | 9 | 15 | 5 |
| | *SOT* | 3 | 4 | 9 | 2 |

Supplementary Table 28

**Target Identification cross validation:**
**Participant 8**

| | | *Classification* | |
|---|---|---|---|
| | | *CTI* | *FTI* |
| *True label* | *CTI* | 36 | 8 |
| | *FTI* | 7 | 20 |

Supplementary Table 29

**Movement action test set:**
**Participant 8**

| | | *Classification* | | | |
|---|---|---|---|---|---|
| | | *TT* | *TR* | *MFFT* | *SOT* |
| *True label* | *TT* | 11 | 6 | 3 | 3 |
| | *TR* | 5 | 4 | 0 | 3 |
| | *MFFT* | 1 | 3 | 3 | 0 |
| | *SOT* | 0 | 1 | 1 | 1 |

Supplementary Table 30

**Target Identification test set:**
**Participant 8**

| | | *Classification* | |
|---|---|---|---|
| | | *CTI* | *FTI* |
| *True label* | *CTI* | 7 | 1 |
| | *FTI* | 2 | 3 |

Supplementary Table 31

**Movement action cross validation:**
**Participant 9**

| | | *Classification* | | | |
|---|---|---|---|---|---|
| | | *TT* | *TR* | *MFFT* | *SOT* |
| *True label* | *TT* | 44 | 25 | 26 | 28 |
| | *TR* | 15 | 33 | 11 | 6 |
| | *MFFT* | 17 | 7 | 19 | 9 |
| | *SOT* | 7 | 5 | 9 | 6 |

Supplementary Table 32

**Target Identification cross validation:**
**Participant 9**

| | | *Classification* | |
|---|---|---|---|
| | | *CTI* | *FTI* |
| *True label* | *CTI* | 23 | 14 |
| | *FTI* | 10 | 17 |

Supplementary Table 33

**Movement action test set:**
**Participant 9**

| | | *Classification* | | | |
|---|---|---|---|---|---|
| | | *TT* | *TR* | *MFFT* | *SOT* |
| *True label* | *TT* | 8 | 4 | 5 | 5 |
| | *TR* | 3 | 8 | 0 | 0 |
| | *MFFT* | 2 | 2 | 3 | 2 |
| | *SOT* | 2 | 0 | 2 | 1 |

Supplementary Table 34

**Target Identification test set:**
**Participant 9**

| | | *Classification* | |
|---|---|---|---|
| | | *CTI* | *FTI* |
| *True label* | *CTI* | 4 | 3 |
| | *FTI* | 1 | 4 |

Supplementary Table 35

Movement action cross validation:
Participant 10

| | | Classification | | | |
|---|---|---|---|---|---|
| | | *TT* | *TR* | *MFFT* | *SOT* |
| *True label* | *TT* | 76 | 20 | 26 | 22 |
| | *TR* | 10 | 52 | 6 | 8 |
| | *MFFT* | 11 | 6 | 11 | 12 |
| | *SOT* | 3 | 3 | 10 | 4 |

Supplementary Table 36

Target Identification cross validation:
Participant 10

| | | Classification | |
|---|---|---|---|
| | | *CTI* | *FTI* |
| *True label* | *CTI* | 41 | 14 |
| | *FTI* | 8 | 17 |

Supplementary Table 37

Movement action test set:
Participant 10

| | | Classification | | | |
|---|---|---|---|---|---|
| | | *TT* | *TR* | *MFFT* | *SOT* |
| *True label* | *TT* | 13 | 5 | 5 | 2 |
| | *TR* | 3 | 8 | 1 | 1 |
| | *MFFT* | 2 | 1 | 2 | 2 |
| | *SOT* | 0 | 0 | 0 | 3 |

Supplementary Table 38

Target Identification test set:
Participant 10

| | | Classification | |
|---|---|---|---|
| | | *CTI* | *FTI* |
| *True label* | *CTI* | 6 | 4 |
| | *FTI* | 1 | 3 |
